# The So-Called Third Heart-Sound

**Published:** 1908-11-07

**Authors:** 


### THE SO-CALLED THIRD HEART-SOUND.

When there is reduplication of either the first or the second cardiac sound there is, in a sense, a third sound; neither this reduplication, however, nor any of the recognised cardiac bruits is meant by the term "the third heart-sound." In a certain number of perfectly healthy persons, especially young persons, there is to be heard at the cardiac impulse a slight third sound, which occurs early in diastole shortly after the second sound. The chief importance of this in every-day practice is in connection with candidates who come up to pass a medical examination for life insurance, for a clerkship, for one of the Services, or for something of this kind. Are the hearts which produce a third heart-sound in this way normal or not? Apparently there is no sharp line of demarcation between what may be termed a normal third heart-sound on the one hand and an abnormal third heart-sound on the other. When the case is before one it will be comparatively easy to assign the proper value to what one hears; but Dr. Thayer, who with Dr. MacCallum has done a considerable amount of experimental work in connection with cardiac murmurs, puts forward the suggestion that it is merely an exaggeration of the healthy third heart-sound that gives the character to the morbid triple sound of the gallop rhythm of pericarditis, and to the peculiar diastolic third sound and shock that is to be noted in some cases of adherent pericardium.

Gibson, Hirschfelder, and other observers have paid considerable attention to this third heart-sound, studying it by means of venous pulse tracings, cardiograms, and so forth. The result of their investigations seems to show that the early diastolic sound sometimes heard in normal hearts is closely associated with the rapid entrance of the blood into the ventricle at the onset of diastole. At this period, early in the diastole, some structure is thrown into sufficient tension to produce audible vibrations; this structure is very likely the mitral valve; at the same time there is some sudden, slight, temporary arrest or interruption or delay in the rapid dilatation of the ventricle. Possibly the moment at which this occurs is that at which the hypothetical active dilatation of the ventricle merges into its passive expansion by the inpouring blood. That an interruption in the ventricular diastole simultaneous with the occurrence of the third cardiac sound does actually occur in some cases was ocularly demonstrated in one of Dr. Thayer's dogs, in which a visible interruption of the dilatation of the ventricle was observed in association with the sound.

Be this as it may, a slight third sound which is not pathological may sometimes be heard at the impulse of the heart in diastole shortly after the second sound. The sound is commoner with a slow pulse; it is better heard when the patient is lying down than when he is standing or sitting, and best of all in the left lateral decubitus; it is sometimes much more audible during expiration than it is during inspiration; the sound is not infrequently associated with a shock, which may be visible as well as palpable; and the condition is not imaginary nor an artefact, for the sound is represented by a vibration upon both the cardiogram and the jugular vein pulse tracing.

